# Retraction Note: Lnc-PDZD7 contributes to stemness properties and chemosensitivity in hepatocellular carcinoma through EZH2- mediated ATOH8 transcriptional repression

**DOI:** 10.1186/s13046-025-03364-0

**Published:** 2025-03-21

**Authors:** Yi Zhang, Bo Tang, Jun Song, Shuiping Yu, Yang Li, Huizhao Su, Songqing He

**Affiliations:** 1 Department of Hepatobiliary Surgery, The First Affiliated Hospital of Guangxi Medical University, No.6 Shuangyong Road, Nanning, 530021 Guangxi People’s Republic of China; 2 Department of General Surgery, Affiliated hospital of Xuzhou Medical University, Xuzhou, 221000 China

**Retraction Note:** ***J Exp Clin Cancer Res*** **38, 92 (2019)**

10.1186/s13046-019-1106-2

The Editor-in-chief has retracted this article. Following this article’s publication, concerns were raised regarding similarities of images within the article and previously published article [1]. The authors were unable to provide raw data for further validation upon request.

The Editor-in-Chief therefore no longer has confidence in the results and conclusions presented in this study.

- Similarities between 2F and 6G;
- Similarities between 2G and 4F;
- Similarities between 3B and 3F;
- Similarities between 4G and 6G;
- Similarities of 3C and 3G with Fig. 8A of [1].

Bo Tang, Yi Zhang, and Songqing He agree to this retraction. Jun Song did not respond to the editor/Publisher regarding this retraction. The Publisher is unable to find the current email addresses of Yang Li, Shuiping Yu and Huizhao Su.

## References

[1] Retracted Article: Tang, B., Li, Y., Yuan, S., Tomlinson, S., & He, S. (2013). Upregulation of the δ opioid receptor in liver cancer promotes liver cancer progression both in vitro and in vivo Retraction in /10.3892/ijo.2023.5504. International Journal of Oncology, 43, 1281–1290. 10.3892/ijo.2013.2046 10.3892/ijo.2013.2046 23903826

